# Zurletrectinib is a next-generation TRK inhibitor with strong intracranial activity against *NTRK* fusion-positive tumours with on-target resistance to first-generation agents

**DOI:** 10.1038/s41416-024-02760-1

**Published:** 2024-06-20

**Authors:** Paola Roa, Valentina Foglizzo, Guilherme Harada, Matteo Repetto, Amanda Kulick, Elisa de Stanchina, Michelle de Marchena, Supipi Auwardt, Shaza Sayed Ahmed, Nicole Virginia Bremer, Soo-Ryum Yang, Yangbo Feng, Chao Zhou, Norman Kong, Ruixia Liang, Haipeng Xu, Bin Zhang, Alberto Bardelli, Eneda Toska, Andrea Ventura, Alexander Drilon, Emiliano Cocco

**Affiliations:** 1 Department of Biochemistry and Molecular Biology, University of Miami, Miller School of Medicine, Miami, FL USA; 2 Sylvester Comprehensive Cancer Center (SCCC), Miami, FL USA; 3 Department of Medicine, Memorial Sloan Kettering Cancer Center, New York, NY USA; 4 Department of Oncology and Haemato-Oncology, University of Milan, 20133 Milan, Italy; 5 Antitumor Assessment Core Facility, Memorial Sloan Kettering Cancer Center, New York, NY USA; 6 Department of Pathology, Memorial Sloan Kettering Cancer Center, New York, NY USA; 7 Department of Molecular and Cellular Pharmacology, University of Miami Miller School of Medicine, Miami, FL USA; 8 InnoCare Pharma Limited, Beijing, China; 9 Department of Oncology, Molecular Biotechnology Center, University of Torino, Torino, Italy; 10 IFOM-ETS, The AIRC Institute of Molecular Oncology, Milan, Italy; 11 Department of Oncology, Sidney Kimmel Comprehensive Cancer Center, Baltimore, MD USA; 12 Department of Biochemistry and Molecular Biology, Johns Hopkins School of Public Health, Baltimore, MD USA; 13 Cancer Biology and Genetics Program, Memorial Sloan Kettering Cancer Center, New York, NY USA; 14 Department of Medicine, Weill Cornell Medical College, New York, NY USA

**Keywords:** Oncogenes, Cancer

## Abstract

**Background:**

While *NTRK* fusion-positive cancers can be exquisitely sensitive to first-generation TRK inhibitors, resistance inevitably occurs, mediated in many cases by acquired *NTRK* mutations. Next-generation inhibitors (e.g., selitrectinib, repotrectinib) maintain activity against these TRK mutant tumors; however, there are no next-generation TRK inhibitors approved by the FDA and select trials have stopped treating patients. Thus, the identification of novel, potent and specific next-generation TRK inhibitors is a high priority.

**Methods:**

In silico modeling and in vitro kinase assays were performed on TRK wild type (WT) and TRK mutant kinases. Cell viability and clonogenic assays as well as western blots were performed on human primary and murine engineered *NTRK* fusion-positive TRK WT and mutant cell models. Finally, zurletrectinib was tested in vivo in human xenografts and murine orthotopic glioma models harboring TRK-resistant mutations.

**Results:**

In vitro kinase and in cell-based assays showed that zurletrectinib, while displaying similar potency against TRKA, TRKB, and TRKC WT kinases, was more active than other FDA approved or clinically tested 1^st^- (larotrectinib) and next-generation (selitrectinib and repotrectinib) TRK inhibitors against most TRK inhibitor resistance mutations (13 out of 18). Similarly, zurletrectinib inhibited tumor growth in vivo in sub-cute xenograft models derived from *NTRK* fusion-positive cells at a dose 30 times lower when compared to selitrectinib. Computational modeling suggests this stronger activity to be the consequence of augmented binding affinity of zurletrectinib for TRK kinases. When compared to selitrectinib and repotrectinib, zurletrectinib showed increased brain penetration in rats 0.5 and 2 h following a single oral administration. Consistently, zurletrectinib significantly improved the survival of mice harboring orthotopic *NTRK* fusion-positive, TRK-mutant gliomas (median survival = 41.5, 66.5, and 104 days for selitrectinib, repotrectinib, and zurletrectinib respectively; *P* < 0.05).

**Conclusion:**

Our data identifies zurletrectinib as a novel, highly potent next-generation TRK inhibitor with stronger in vivo brain penetration and intracranial activity than other next-generation agents.

## Background

Tropomyosin receptor kinases A, B, and C (TRKA, TRKB, and TRKC) constitute a family of transmembrane glycoproteins which are encoded by the neurotrophic tyrosine receptor kinase genes 1, 2, and 3, respectively (*NTRK1*, *NTRK2*, and *NTRK3*) [1]. These receptor tyrosine kinases are high affinity receptors for neurotrophins, growth factors which play a crucial role during neurogenesis [2]. Neurotrophin binding leads to TRK receptor dimerization which promotes the autophosphorylation of tyrosine residues within the activation loop of the kinase domain [3]. These phosphorylated tyrosines serve as docking sites for adapters and downstream effectors that ultimately lead to the activation of the RAS/MAPK, PI3K, and PLC_γ_ pathways [4].

Gene fusions involving the *NTRK*1-3 genes have been identified as oncogenic drivers in various cancer types [5–8]. While their frequency is low (less than 1%) in common histologies such as breast, colorectal, and lung cancers, they are considered pathognomonic (occurring in >90% of cases) in rare cancers such as mammary analog secretory carcinomas and infantile fibrosarcoma [5, 9]. These oncogenes promote the synthesis of TRK fusion chimeric proteins that are aberrantly expressed and constitutively active in a ligand independent manner.

Importantly, *NTRK* fusion-positive tumors are highly sensitive to TRK inhibitors. Responses to the 1^st^-generation TRK inhibitors entrectinib and larotrectinib were reported in up to 80% of patients in a histologic agnostic manner [10, 11]. These results led to the approval of these drugs by various regulatory agencies for the treatment of adult and pediatric *NTRK* fusion-positive tumors [12]. Despite the substantial efficacy of these 1^st^-generation agents, resistance eventually develops, with the most frequently reported cases being on-target [13, 14]. The most common resistance mutation that emerges following 1^st^-generation drug treatment is the solvent front substitution (TRKA G595R/TRKC G623R) that generates steric hindrance, thus impairing drug binding [13, 14]. The emergence of this and other on-target mutations (e.g., the TRKA F589L/TRKC F617L gatekeeper substitution or the TRKA G667C/TRKC G696C xDFG mutation) was predicted and thus, next-generation TRK inhibitors such as selitrectinib and repotrectinib were developed alongside 1^st^-generation drugs to overcome resistance [15–18].

Resistance to next-generation drugs has also been described. Similar to what has been observed with 1^st^-generation drugs, this can be off-target (mainly through the activation of the MAPK pathway [19]) or on-target [20]. In the latter, the emergence of TRK xDFG single or compound mutations, which hinder drug binding, is the only mechanism known [20]. These mutations, while conferring resistance to 1^st^- and next-generation type I TRK inhibitors, are exquisitely sensitive to type II multikinase inhibitors known to also target TRK kinases [20].

Zurletrectinib is a recently developed novel next-generation TRK inhibitor that showed activity against TRK WT and mutant kinases [21]. This study aims at characterizing the in vitro and in vivo activity of zurletrectinib against TRK kinases and *NTRK* fusion-positive primary and engineered models harboring TRK resistance mutations.

## Methods

### Compounds

Larotrectinib, selitrectinib, repotrectinib and zurletrectinib were obtained from InnoCare. Cabozantinib was purchased from MedChem Express. All drugs were dissolved in DMSO to produce 10 mM stocks and stored at −20 °C. Cabozantinib was added to our studies because it was previously reported to be specifically active against TRK xDFG mutant tumors [20, 22].

### Cell lines

*NTRK* fusion-positive colorectal cancer cell lines IRC, Kor1, and KM12 were utilized. The IRC and the KM12 cell lines were obtained from Dr. Bardelli and cultured according to the described protocols [14, 19, 23]. The Kor1 cell line was obtained from Lee et al. [24] and cultured on laminin pre-treated plates in RPMI media supplemented with 10% Fetal Bovine Serum (FBS). All cell lines were authenticated by STR and periodically screened for the presence of mycoplasma. For the mutant models, two human-derived colorectal cancer cell lines (IRC) and two mouse-derived glioma models (NLS) were used. The single mutant IRC *LMNA-NTRK1* TRKA G595R primary cell line was obtained from Dr. Bardelli and established from the tumor of a patient who had progressed on earlier generation TKI treatment (the specimen was used to generate a Patient-Derived-Xenograft from which the cell line was then established) [14]. The double mutant IRC *LMNA-NTRK1* TRKA G595R/G667C cell line was established following chronic exposure of the single mutant patient-derived cell line to increasing concentrations of repotrectinib. Sequencing of the double mutant cell line was performed using MSK-IMPACT [25]. IRC primary cell lines were cultured with DMEM/F12 50:50 Mix (Corning) supplemented with 10% FBS. NLS *Bcan-Ntrk1* Trka G598R and *Bcan-Ntrk1* Trka G670C single mutants as well as double mutants NLS *Bcan-Ntrk1* Trka G598R/G670A and NLS *Bcan-Ntrk1* Trka G598R/G670C isogenic models were established using CRISPR/Cas9 to knock-in TRKA solvent-front and xDFG mutations into tumor cells derived from a *Bcan-Ntrk1*-driven glioma mouse model. Trka G598R is the mouse ortholog to human TRKA G595R while Trka G670A and Trka G670C are the mouse ortholog to human TRKA G667A and TRKA G667C, respectively. Mouse p53 ^−^/^−^ *Bcan-Ntrk1* glioma cells were plated on laminin-coated plates and cultured with Neurocult Stem Cell Basal Media with Proliferation Supplements (Stem Cell Technologies) [26]. Ba/F3 cell lines stably expressing WT and mutant versions (i.e. G595R, G667C/A, V573M, F589L, F633L, F617L, G623R/E, G639R, G696A/C, G709C, V608D/M) of human TRKA, TRKB or TRKC were generated using either lentiviral transduction or standard transfection methods by a contract research organization (Kyinno, Beijing, China).

### Drug screenings

CellTiter-Glo Cell Viability Assays (Promega) and crystal violet clonogenic assays were performed on primary *NTRK* fusion-positive colorectal cancer cell lines, mouse Ba/F3 cell lines and isogenic mouse glioma cell lines. For the CellTiter-Glo assays, three biological replicates were performed, with each condition being assayed in triplicate determinations [22]. Cells were seeded in a 96-well plate in the afternoon at optimal density. The following morning, larotrectinib, selitrectinib, repotrectinib, zurletrectinib and cabozantinib (1:2, 1:3 or 1:4 dilutions with a maximum concentration of 10 µM) were added. Plates were removed from the incubator 72 h later and CellTiter-Glo reagent was added. Absorbance was read at 490 nm in accordance with Promega’s protocol. Data is presented as a survival percentage on the y-axis (mean ± STDEV) normalized to the control DMSO-treated cells deemed 100% viable. Drug concentrations on the x-axis are represented as a base 10 logarithm (LOG). For the crystal violet assays, cells were seeded in a 24-well plate at optimal densities in the afternoon. The following morning, larotrectinib, selitrectinib, repotrectinib and zurletrectinib were added at concentrations ranging from 1 nM to 5000 nM. Crystal violet plates were removed from the incubator 72 h later, washed with PBS, fixed with 4% paraformaldehyde for 15 min, and stained with crystal violet for 10 min. Crystal violet was washed off and plates were left to dry prior to imaging. Clonogenic assays were performed in three biological replicates.

### Antibodies and western blot

Cell lines were seeded in six-well plates in the afternoon at 600,000 cells per well in full media. The following morning, cells were treated with 100 nM of each compound for 30 min. For isogenic mouse glioma cell lines *Bcan-Ntrk1* Trka G598R and *Bcan-Ntrk1* Trka G598R/G670A cells were seeded in six-well plates in the afternoon at 800,000 cells per well in full media. The following evening, cells were put into starvation with Neurocult Basal media containing only penicillin and streptomycin. The following morning, cells were treated with 100 nM of each compound for 30 min. Upon completion of the 30 min treatment total protein lysates were extracted in 75 uL of radioimmunoprecipitation assay (RIPA) buffer containing phosphatase and protease inhibitors. Lysates were quantified using bicinchoninic acid in accordance with the manufacturer’s protocol. Lysates were separated with sodium dodecyl sulfate-polyacrylamide gel electrophoresis gels in accordance with standard methods. All membranes were probed with the following antibodies: phospho-p44/42 MAPK (Erk1/2; T202/Y204) clone D13.14.4E (4370S, Cell Signaling Technology); total ERK1/2 (9102S, Cell Signaling Technology); pan TRK clone A7H6R (92991S, Cell Signaling Technology); phospho-TRKA (Y674/675) clone C50F3 (4621S, Cell Signaling Technology) and β-actin clone 13E5 (4970S, Cell Signaling Technology). Western blots were performed in three biological replicates and a representative western blot is shown.

### Kinase assays

Recombinant kinases were purchased from SignalChem Biotech Inc (Richmond, BC, Canada). In vitro kinase assays were performed following the optimization of the HTRF Kinase Assay (Cisbio) [27]. Compounds larotrectinib, selitrectinib, repotrectinib, and zurletrectinib were tested in 10-dose IC_50_ mode with four-fold serial dilutions starting at 100 nM (for TRKA, TRKB, and TRKC) or 1 or 10 µM (for TRKA mutant kinases). ATP concentrations ranged from 1 to 10 µM based on internal optimization. Substrate concentrations ranged from 1 to 10 µM at Km of each kinase.

### Docking

Evaluation of zurletrectinib’s chemical structure revealed numerous similarities with the related macrocyclic, pyrazolopyrimidine, pan-TRK, DFG-Din, Alpha-Cin, and Activation-Loop-in ATP-competitive inhibitor, repotrectinib. We accessed the Protein Data Bank (PDB; https://www.rcsb.org/) and retrieved available crystal structures of the wild-type TRKA-Repotrectinib complex (PDB ID: 7VKO) and the solvent-front mutant TRKA G595R (PDB ID: 7VKN). Unfortunately, no TRKB or TRKC DFG-Din plus Alpha-Cin crystal structures were found on PDB suitable for zurletrectinib docking. We thus modeled these from FASTA sequences using SWISS-MODEL (http://swissmodel.expasy.org) [28] homology modeling starting from TRKA 7VKO as a template. To examine the influence of secondary mutations on the zurletrectinib-TRK interaction, we generated several solvent-front and xDFG mutant proteins using Pymol (http://pymol.org) [29]. Before docking, proteins were prepared using the Protein Preparation Wizard in Schrödinger’s Maestro. We obtained the chemical structure of zurletrectinib from PubChem and prepared it using Maestro’s LigPrep function (https://www.schrodinger.com/life-science/learn/white-papers/protein-preparation-wizard/). The receptor grid was centered on catalytic spine 6 (CS6) residue (TRKA L656, TRKB L699, TRKC L686), which is located in the center of the ATP binding pocket. A cubic grid box of dimensions 30 Å × 30 Å × 30 Å was constructed. Docking constraints were set to ensure the generation of a hydrogen bond with the peptide backbone of the hinge’s third (H3) residue (TRKA M593, TRKB M636, TRKC M620). We executed ligand docking simulations using the Glide module in standard precision mode [30], with the hydrogen-bond generation with H3 residue set as a constraint. The highest-ranking poses were visually inspected in Maestro and juxtaposed with the related macrocyclic inhibitor, repotrectinib. We analyzed interactions, such as hydrogen bonds and hydrophobic contacts for both WT and secondary mutant proteins.

### In vivo efficacy studies

KM12 and Ba/F3 *LMNA-NTRK1*, TRKA G595R xenografts were generated by subcutaneous injection of five and six million cells into the right flank of 6–8 weeks old BALB/c nude female mice, respectively. Treatment with zurletrectinib, selitrectinib and larotrectinib started at tumor reaching around 150 mm^3^. Xenografts were randomized into different groups (8 mice per group) based on tumor size and dosed orally with zurletrectinib (0.1, 0.3, 1 mg/kg BID for KM12 models and 0.3, 1, 3 mg/kg BID for Ba/F3 models), selitrectinib (30 mg kg^−1^ BID per day) and larotrectinib (30 mg kg^−1^ BID) for constant 10 days or 23 days for KM12 or Ba/F3 models, respectively. Eight mice per group were included in each experiment. Randomization was conducted without utilizing a blind method. Tumors were measured twice weekly using calipers and body weight was also assessed twice weekly. At the end of each treatment, animals were euthanized. The vehicles were formulated as follows: were first dissolved in Cremophor EL/ethanol (50:50) as a 4× stock solution, vortexed, sonicated and then diluted to a 1× solution with saline before use.

### Brain distribution in rats

All pharmacodynamics (PK) studies in rats were conducted according to protocols approved by the Animal Care and Use Committee at InnoCare pharmacology company in China. Male Sprague Dawley (SD) rats (*n* = 3 per time point) received a single oral administration of 10 mg/kg of zurletrectinib, selitrectinib and repotrectinib. Zurletrectinib was suspended in 0.5% methylcellulose; repotrectinib and selitrectinib were reconstituted in 0.5% methylcellulose containing 10% Cremophor EL. For the collection of plasma, brain tissue and cerebrospinal fluid (CSF), animals were euthanized with CO_2_ at 0.5 and 2 h post dosing and blood samples were in EDTA-treated microtubes. Brain tissues were homogenized and immediately flash frozen (−80 °C). Blood samples and CSF were spun at 2000 × *g* for 15 min at 4 °C, and plasma was removed and stored at −80 °C until liquid chromatography-tandem mass spectrometry (LC-MS/MS) analysis. The agents in rat plasma, brain homogenate or CSF samples were extracted and quantified by LC-MS/MS.

### Intracranial efficacy studies

In vivo brain orthotopic experiments were carried out at Memorial Sloan Kettering Cancer Center (MSKCC) [20]. Two mouse *Bcan-Ntrk1* glioma models were used: single solvent front mutant Trka G598R (clone 1) and double xDFG mutant Trka G598R/G670A (clone 3). For each model, five arms were used with six mice per arm, totaling 60 mice. Athymic female mice (6–8 weeks old) were anesthetized with ketamine/xylazine and administered a preoperative dose of buprenorphine prior to stereotactic intracranial injection. Each mouse was injected with 200,000 cells in a volume of 2 uL 3 mm deep, 1 mm to the right of the sagittal suture (midline), and 1 mm posterior to the coronal suture behind the bregma with the aim being to target the right lateral ventricle. One week post cell implantation, the mice were randomized into different treatment groups and treatments began. The drugs utilized were larotrectinib, selitrectinib, repotrectinib and zurletrectinib. The vehicles were as follows: 100% labrafac for larotrectinib, 1% CMC, 0.5% Tween80 for selitrectinib, 0.5% methyl cellulose for repotrectinib, and zurletrectinib was first dissolved in Cremophor EL/ethanol (50:50) as a 4× stock solution, vortexed, sonicated and then diluted to a 1× solution with saline. For both the Trka G598R model and the Trka G598R/G670A model, mice were treated five days a week BID with the larotrectinib and selitrectinib groups receiving doses of 30 mg/kg and the repotrectinib and zurletrectinib groups receiving doses of 15 mg/kg. Mouse weights were monitored twice a week and survival was recorded and graphed on GraphPad Prism 9 [31, 32].

### Statistical analysis

Statistical analyses were conducted using GraphPad Prism 9 (GraphPad Software Inc.). For CellTiter-Glo assays, raw data obtained were normalized to the DMSO control utilizing Microsoft Excel. GraphPad Prism 9 was used to determine the 50% growth inhibition concentration (IC_50_) using non-linear regression and curve fitting [22, 31]. Data is presented as the mean ± standard deviation (STDEV) of all replicates. Measurements were assessed for normal distribution and homogeneity of variance using appropriate tests and were analyzed using analysis of variance or T-Tests, and nonparametric tests were used for non-normal distributed data. Animal studies utilized five arms with six or eight mice per arm. A Kaplan–Meier test was used to assess lifespan characteristics and for comparison between groups [33]. Median survivals were obtained using GraphPad Prism 9. Sample size was based on statistical power analysis to ensure sufficient capability to detect a pre-specified effect size. We assumed a moderate effect size, with an alpha level set at 0.05, and a power (1-β) set at 0.80. *P* values were calculated using a Log-rank or Mantel–Cox test using GraphPad Prism 9. A *P* value < 0.05 was considered statistically significant.

## Results

### Zurletrectinib is active against TRK kinases and *NTRK* fusion-positive tumors

**Fig. 1:**
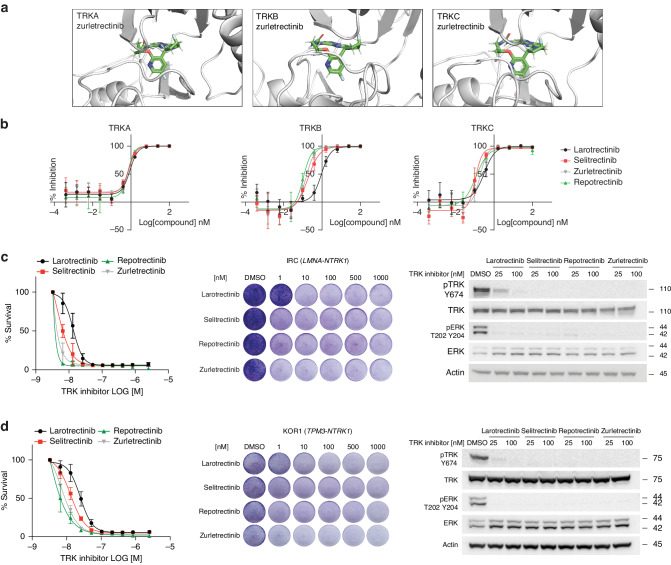
Activity of zurletrectinib against TRK kinases. **a** Molecular docking and **b** in vitro kinase assays showing binding and activity of zurletrectinib against TRKA, TRKB, and TRKC kinases. Kinase assays were run in duplicates. Data were analyzed using GraphPad Prism 9 and are presented as % of inhibition normalized to untreated samples (mean ± STDEV). Groups were compared using a paired Student’s T-Test. *P* < 0.05 was considered statistically significant. **c**, **d** Proliferation and clonogenic assays, and western blot analyses following treatment of two primary *NTRK* fusion-positive cell lines with zurletrectinib. The activity of zurletrectinib was compared against the activity of other 1^st^- (larotrectinib) or next-generation (selitrectinib and repotrectinib) TRK inhibitors (**b–d**).

The binding of zurletrectinib to TRKA, TRKB, and TRKC was evaluated using molecular docking. Similar to other type I TRK inhibitors, zurletrectinib occupies the ATP binding pocket of the TRK kinases and interacts with key residues at the gatekeeper position (TRKA F589/TRKB F633/TRKC F617) and at the xDFG position (TRKA G667/TRKB G709/TRKC 696) (Fig. 1a and Supplementary Fig. 1). Importantly, and unlike other TRK inhibitors approved or in clinical development, through its fluoropyrrolidine moiety, zurletrectinib makes unique interactions with the K544 and D668 residues of TRKA (or with the paralogue residues of TRKB and TRKC) (Supplementary Fig. 1) which are predicted to endow zurletrectinib with high selectivity for TRK kinases. To test the activity of zurletrectinib against TRKA, TRKB, and TRKC recombinant kinases, we performed in vitro kinase assays. IC_50_s calculated for zurletrectinib against TRKA, TRKB, and TRKC were 0.81 nM, 0.145 nM, and 0.184 nM, respectively. These values were lower than IC_50_s obtained with larotrectinib against the same recombinant TRK kinases, but similar to the ones obtained with selitrectinib and repotrectinib (Fig. 1b and Supplementary Fig. 2a). Consistently, zurletrectinib, selitrectinib, and repotrectinib similarly inhibited the growth and TRK-mediated signaling of primary human cancer cell lines harboring *NTRK* fusions. Specifically, we tested the activity of zurletrectinib against two primary colorectal cancer (CRC) cell lines harboring a *LMNA-NTRK1* fusion (IRC-I-XL [14]), or a *TPM3-NTRK1* fusion (Kor1 [24]). IC_50_s calculated for zurletrectinib against the IRC-I-XL and the Kor1 cell lines were 0.47 nM and 7.2 nM, respectively. These values were significantly lower than IC_50_s calculated for larotrectinib against the same models (IC_50_s of 13.4 nM and 24.8 nM, respectively; *P* < 0.05) but comparable to the ones obtained with the next-generation agents selitrectinib (IC_50_s of 4.6 nM and 13 nM, respectively) and repotrectinib (IC_50_s of 1.6 nM and 2.4 nM, respectively) (Fig. 1c, d and Supplementary Fig. 2b, c).

### Zurletrectinib is active against 1^st^-generation TRK inhibitor resistance mutations in vitro

**Fig. 2:**
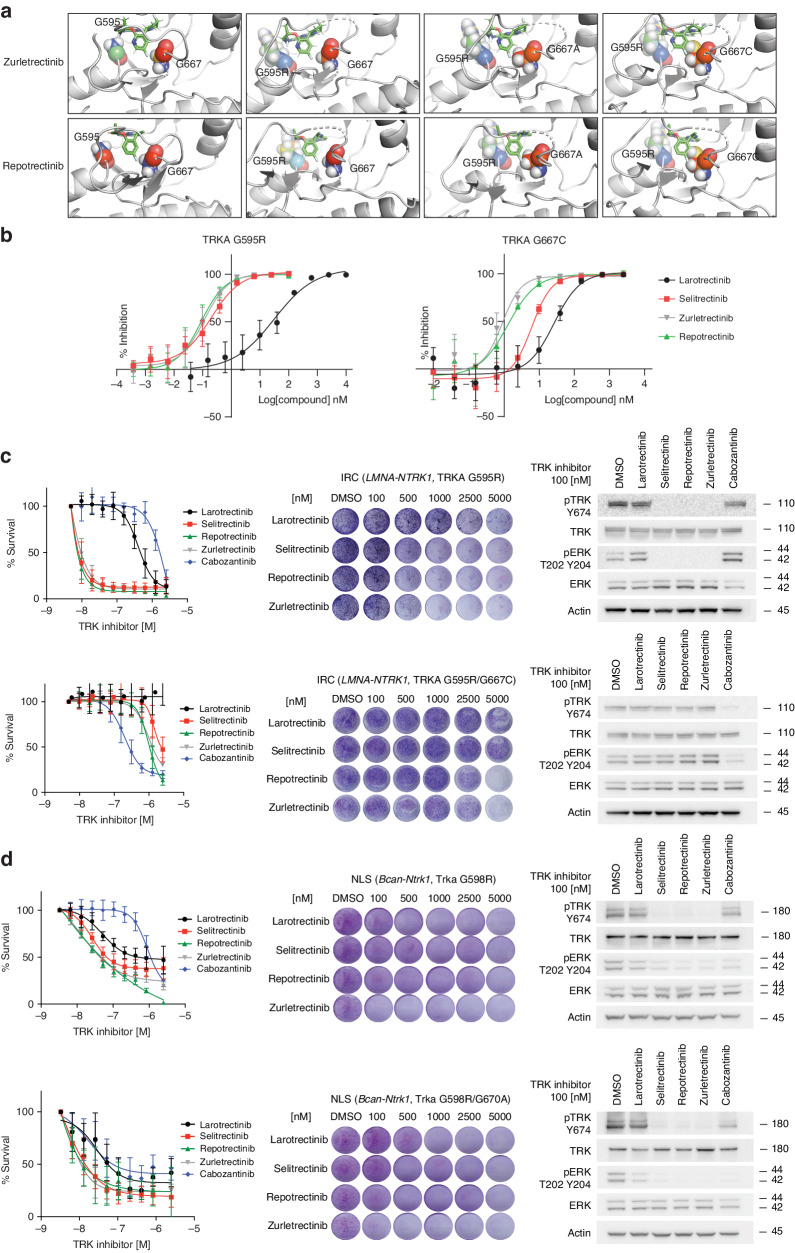
Activity of zurletrectinib against solvent front and xDFG TRK mutant kinases in vitro. **a** Molecular docking and **b** in vitro kinase assays showing binding and activity of zurletrectinib against TRKA mutant kinases. Data were analyzed using GraphPad Prism 9 and are presented as % of inhibition normalized to untreated samples (mean ± STDEV). Groups were compared using a paired Student’s T-Test. *P* < 0.05 was considered statistically significant. Proliferation and clonogenic assays, and western blot analyses following treatment of two human primary (**c**) and two engineered mouse (**d**) *NTRK* fusion-positive cancer cell lines harboring TRK resistance mutations with zurletrectinib. The activity of zurletrectinib was compared against the activity of other 1^st^- (larotrectinib) or next-generation (selitrectinib and repotrectinib) TRK inhibitors (**b**–**d**).

Selitrectinib and repotrectinib are both next-generation TRK inhibitors specifically developed to maintain activity against on-target mutations that are acquired on progression to 1^st^-generation TRK inhibitors. Thus, we tested whether zurletrectinib is also active against these resistant mutations. To do so, we first selected the well characterized TRKA G595R solvent front mutation as well as the TRKA G667A and TRKA G667C xDFG mutations that have been identified as single or compound mutations in tumors of patients progressing on 1^st^- or next-generation TRK inhibitors [11, 14, 19, 20] (Supplementary Fig. 3). In order to predict the binding of zurletrectinib to the different mutants, we utilized in silico modeling. Our predictions showed that zurletrectinib maintains binding capacity against TRKA G595R, and the G667A mutant but less so against the TRKA xDFG mutant harboring the relatively bulky G667C substitution (Fig. 2a). This is likely due to the steric hindrance that cysteine generates with the fluoropyrimidine-containing moiety of zurletrectinib. The same results were obtained when we modeled repotrectinib in complex with the same TRKA mutant kinases.

To validate our prediction, we then generated TRKA G595R and G667C recombinant kinases and tested the activity of zurletrectinib, selitrectinib, and repotrectinib in in vitro kinase assays. Consistent with our prediction, while all three next-generation agents were active in the low nanomolar range against TRKA G595R, their potency was significantly reduced against the TRKA G667C xDFG mutant (Fig. 2b [20]). In agreement with our in vitro data, zurletrectinib, selitrectinib, and repotrectinib were able to inhibit the growth and TRKA-mediated signaling in primary human *NTRK* fusion-positive cell lines with the TRKA G595R mutation, but not with the TRKA G595R/G667C compound mutation (Fig. 2c and Supplementary Fig. 4b, c), which was instead particularly sensitive to the type II TRK inhibitor cabozantinib, that we used as control [20]. Similarly, when we tested selitrectinib, repotrectinib, and zurletrectinib against mouse glioma *Bcan-Ntrk1* fusion-positive cell lines engineered by CRISPR to harbor the mouse orthologue single and double Trka resistant mutations (Trka G598R, Trka G670C, Trka G598R/G670A, and Trka G598R/G670C, respectively), all were highly active against the Trka G598R, and the Trka G598R/G670A but less so against the Trka G670C or the Trka G598R/G670C mutants (Fig. 2d, Supplementary Figs. 4b, c, and 5).

Next, we tested the activity of zurletrectinib against mouse Ba/F3 cells [34] transduced to express all TRKA, TRKB, and TRKC 1^st^-generation TRK inhibitor resistance mutations described to date (Supplementary Fig. 3) [11, 14, 19, 20]. Cell viability assays performed on these models showed that zurletrectinib was the most powerful next-generation TRK inhibitor against most of these mutants with the exception of the TRKA F589L/TRKB F633L/TRKC F617L gatekeeper mutants for which repotrectinib was more active [15] (Table 1).

**Table 1:** Anti-proliferative activity of zurletrectinib in Ba/F3 cells transduced with WT and mutant *NTRK* fusions.

Cell lines/*NTRK* status	IC_50_ [nM]			
	Larotrectinib	Selitrectinib	Zurletrectinib	Repotrectinib
Ba/F3 *LMNA*-*NTRK*1	21.8	3.38	1.20	2.23
Ba/F3 *ETV6*-*NTRK*2	53.6	9.10	5.61	5.94
Ba/F3 *ETV6*-*NTRK*3	14.2	1.89	1.40	1.46
Ba/F3 *LMNA*-*NTRK1*-F589L	614	46.9	40.8	4.43
Ba/F3 *ETV6*-*NTRK2*-F633L	3113	119	163	11.9
Ba/F3 *ETV6*-*NTRK3*-F617L	893	21.1	31.2	2.41
Ba/F3 *LMNA*-*NTRK1*-G595R	3204	18.1	11.0	16.2
Ba/F3 *ETV6*-*NTRK2*-G639R	3809	81.2	40.0	60.1
Ba/F3 *EVT6*-*NTRK3*-G623R	962	6.70	6.45	11.6
Ba/F3 *ETV6*-*NTRK3*-G623E	61.3	3.23	0.69	3.06
Ba/F3 *LMNA*-*NTRK1*-G667A	118	21.1	5.27	6.48
Ba/F3 *LMNA*-*NTRK1*-G667C	1368	163.6	38.8	68.1
Ba/F3 *LMNA*-*NTRK1*-G667S	3195	537	193	139
Ba/F3 *ETV6*-*NTRK2*-G709C	2788	323	72.6	116
Ba/F3 *ETV6*-*NTRK3*-G696A	45.7	5.10	2.24	2.84
Ba/F3 *ETV6*-*NTRK3*-G696C	780	51.6	15.5	32.2
Ba/F3-*LMNA*-*NTRK1*-V573M	45.4	5.47	2.33	2.22
Ba/F3 *LMNA*-*NTRK1*-A608D	19.7	3.16	1.18	2.12
Ba/F3 *ETV6*-*NTRK2*-V689M	13.9	1.44	1.01	1.24

Together, these data suggest that zurletrectinib is a next-generation TRK inhibitor with stronger activity than other clinically tested agents against TRK inhibitor resistance mutations in vitro.

### Zurletrectinib is highly effective in inhibiting tumor growth in vivo in *NTRK* fusion-positive, TRKA WT or mutant models

**Fig. 3:**
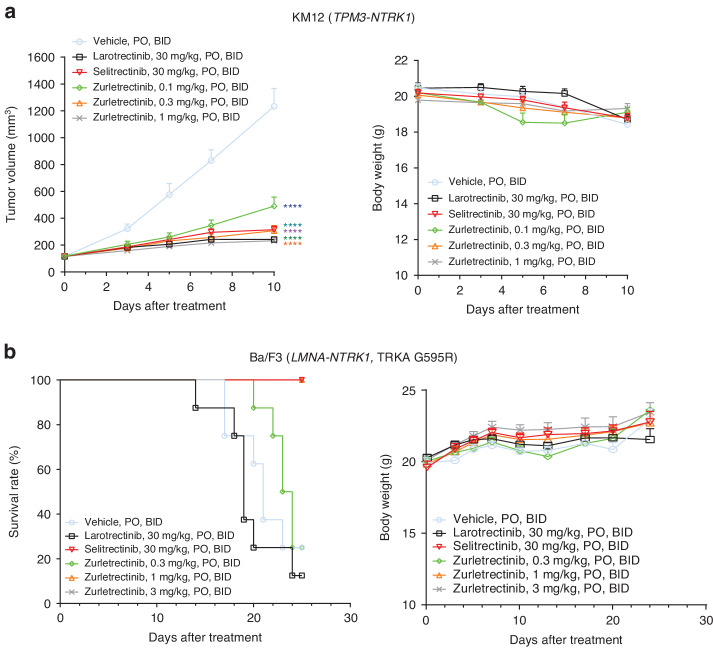
In vivo activity of zurletrectinib in *NTRK* fusion-positive, TRKA WT or mutant xenografts. Activity of zurletrectinib in *NTRK* fusion-positive KM12 xenograft (**a**) and Ba/F3 *LMNA-NTRK1*, TRKA G595R mouse models (**b**). Mice’ weight was recorded over the course of the experiments to monitor treatment related toxicity. Median survivals were obtained using GraphPad Prism 9. *P* values were obtained using a one-way ANOVA [32]. A *P* value < 0.05 was considered statistically significant.

To evaluate the activity of zurletrectinib in vivo, we utilized xenografts derived from the *TPM3-NTRK1* fusion-positive CRC cell line KM12 [23]. Interestingly, while 1^st^- and next-generation TRK inhibitors were similarly potent against this model in vitro (Supplementary Fig. 6), a 30-fold higher dose of larotrectinib and selitrectinib (30 mg/kg BID versus 1 mg/kg BID) was necessary to achieve the same effect on tumor growth inhibition observed with zurletrectinib in vivo. Importantly, no weight loss was reported during the course of the experiment in any of the groups tested (Fig. 3a), suggesting no signs of treatment related toxicity. Having shown the potency of zurletrectinib against TRK inhibitor resistance mutations in vitro, we tested its in vivo activity in xenografts derived from Ba/F3 cells transduced with a *LMNA-NTRK1*, TRKA G595R mutant construct. Similar to the in vivo data we obtained with the KM12 model, a dose of 1 mg/kg of zurletrectinib administrated orally twice a day was as effective as a dose of 30 mg/kg of selitrectinib in inhibiting tumor growth. Again, no weight loss was observed during the course of the experiment, suggesting that the treatments were well tolerated (Fig. 3b).

These data suggest that zurletrectinib is a highly potent and safe next-generation TRK inhibitor with strong in vivo activity against *NTRK* fusion-positive, TRK WT and mutant tumor models.

### Zurletrectinib has potent brain penetration and inhibits the growth of orthotopic mouse glioma xenograft models harboring TRK inhibitor resistance mutations

**Fig. 4:**
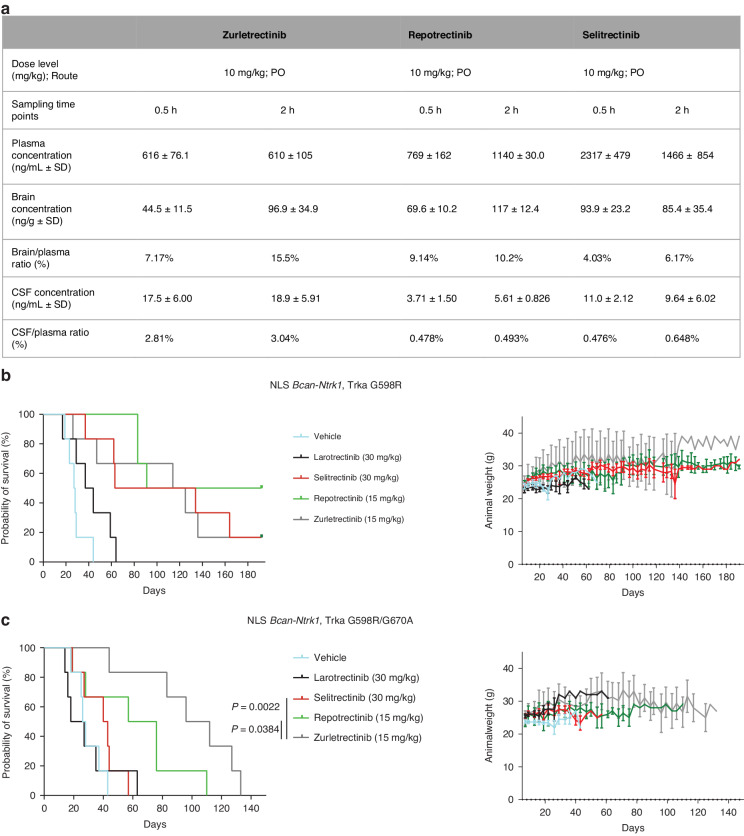
Activity of zurletrectinib in *Ntrk* fusion-positive mouse glioma orthotopic models harboring Trk resistance mutations. Pharmacokinetic studies conducted in rats following a single oral administration (10 mg/kg) of zurletrectinib, repotrectinib, or selitrectinib; CSF (cerebrospinal fluid) (**a**). Survival analysis of mice harboring *Bcan-Ntrk1* (**b**) Trka G598R or *Bcan-Ntrk1* Trka G598R/G670A (**c**) orthotopic gliomas following BID treatment with 30 mg/kg of larotrectinib or selitrectinib, or 15 mg/kg of repotrectinib or zurletrectinib. A Kaplan–Meier test was used for comparison between groups. Median survivals were obtained using GraphPad Prism 9. *P* values were calculated using a Log-rank or Mantel–Cox test using GraphPad Prism 9. Animal weight of *Bcan-Ntrk1* Trka G598R or *Bcan-Ntrk1* Trka G598R/G670A mice was monitored twice a week during the entire course of the experiment.

Patients who progress on 1^st^-generation TRK inhibitors can acquire brain metastases. In addition, *NTRK* fusions have been identified in patients with glioma. Thus, we compared the brain penetration capacity of zurletrectinib, selitrectinib and repotrectinib following their administration into male Sprague Dawley (SD) rats at a single oral dose of 10 mg/kg. Drugs’ concentrations were then measured in brain, cerebrospinal fluid (CSF), and plasma by liquid chromatography-tandem mass spectrometry (LC-MS/MS). All three agents reached the absorption peak at 2 h following treatment (data not shown). At 2 h post-dose, zurletrectinib demonstrates superior brain penetration than repotrectinib and selitrectinib with a brain/plasma ratio of 15.5%, 10.2%, and 6.17%, respectively. Zurletrectinib showed an increase of brain/plasma ratio from 7.17% at 0.5 h to 15.5% at 2 h. Meanwhile, zurletrectinib exhibited the highest CSF/plasma ratio (2.81% and 3.04%) when compared to repotrectinib (0.478% and 0.493%) and selitrectinib (0.476% and 0.648%) at 0.5 and 2 h, respectively (Fig. 4a).

To evaluate whether zurletrectinib increased brain penetration also translated into improved activity against brain tumors compared to the other next-generation agents, we tested the in vivo activity of zurletrectinib in mouse glioma orthotopic xenografts harboring the Trka G598R or the Trka G598R/G670A mutation. One week after intracranial tumor implantation, mice were randomized to receive larotrectinib (30 mg/kg BID), selitrectinib (30 mg/kg BID), repotrectinib (15 mg/kg BID), or zurletrectinib (15 mg/kg BID). Mice were monitored and weighed twice a week for signs of tumor-related discomfort and toxicity and survival was chosen as readout of drug activity. While no significant difference in the survival between selitrectinib, repotrectinib and zurletrectinib was observed in the Trka G598R model (Fig. 4a), zurletrectinib significantly improved survival of mice harboring the Trka G598R/G670A mutation (median survival = 41.5, 66.5, and 104 days for selitrectinib, repotrectinib, and zurletrectinib respectively; *P*(selitrectinib/zurletrectinib = 0.0022; *P*(repotrectinib/zurletrectinib = 0.0384; Fig. 4b). Importantly, no differences in the weight of mice were observed in the different arms, suggesting that the treatments were well tolerated despite some mice having received drugs for more than 150 days (Fig. 4).

## Discussion

In this study, we evaluated the preclinical activity of zurletrectinib, a highly potent next-generation TRK inhibitor. As other next-generation TRK inhibitors tested in the clinic (selitrectinib and repotrectinib), zurletrectinib is a type I agent (Supplementary Fig. 1) [35] which binds to TRK kinases in the ATP binding pocket. While being as active as selitrectinib and repotrectinib against WT TRKA, TRKB, and TRKC kinases, it displays higher potency against most 1^st^-generation TRK inhibitor resistance mutations. These include the TRKA G595R/TRKC G623R solvent front mutations as well as other less common substitutions (Table 1). Importantly, zurletrectinib is also the most potent among the type I next-generation TRK inhibitors that we tested against TRKA G667C/TRKC G696C xDFG mutations (Fig. 2a, b, Table 1, and Supplementary Fig. 5). These mutations, while conferring resistance to the type I TRK inhibitors selitrectinib and repotrectinib, are highly sensitive to type II multikinase agents known to also target TRK kinases (e.g., cabozantinib; Supplementary Fig. 5) [20]. Notably, the clinical use of type II drugs is often limited by the onset of severe adverse effects. In this scenario, the use of zurletrectinib may represent a valid alternative to reestablish disease control while minimizing toxicity.

Importantly, when we tested the in vivo activity of zurletrectinib, we observed that 3 mg/kg of this agent administered orally twice daily in tumor bearing mice was as effective as 30 mg/kg of selitrectinib given at the same regimen (Fig. 3), suggesting zurletrectinib to be a more potent drug in vivo. In addition, when we evaluated the brain penetration of zurletrectinib we found it to be superior than selitrectinib or repotrectinib. Consistently, when we tested the intracranial activity of zurletrectinib against mouse glioma orthotopic models harboring 1^st^-generation TRK inhibitor resistance mutations, we found that zurletrectinib was as efficacious as selitrectinib and repotrectinib against the solvent front single mutant but significantly superior against the Trka G598R/G670A double mutant (Fig. 4). These data suggest that zurletrectinib may be more effective than other next-generation TRK inhibitors in the clinic, especially in patients with brain metastasis [36] or *NTRK* fusion-positive gliomas that harbor TRK xDFG mutations.

A phase I clinical trial to evaluate the safety, tolerability, and pharmacokinetics of zurletrectinib in patients with advanced solid tumors is currently recruiting in the US (NCT05537987) and a phase I/II basket trial evaluating the activity of zurletrectinib in patients with advanced solid tumors or primary central nervous system tumors is ongoing in China (NCT05745623) [37]. Recently reported preliminary results on six patients with *NTRK* fusion-positive tumors showed an overall response rate of 66.7% and a disease control rate of 100%. Importantly, one of these patients who presented with measurable brain metastasis experienced a shrinkage of the targeted brain lesion from 10 to 3 mm [37]. Final results of these trials are expected to be reported in 2024 and 2026, respectively.

While these trials were not specifically designed to test the activity of zurletrectinib against TRK secondary-mutant tumors, patients that develop resistance to 1^st^-generation TRK inhibitors are included in the studies and our data suggest that they may benefit from treatment. It will be very interesting to define mechanisms of resistance to zurletrectinib as it is given upfront to patients whose tumors do not harbor mutated *NTRK* fusions. To this aim, sequencing of liquid or tissue biopsies at the time of progression will be crucial [38, 39]. It will also be important to evaluate response rates and the duration of responses to zurletrectinib in these patients. It is possible that, as it has been observed with alectinib, brigatinib, and lorlatinib in ALK fusion-positive tumors [40, 41], upfront treatment with a next-generation TRK inhibitor may lead to significantly improved outcomes when compared to treatment with an early generation agent. Interestingly, other next-generation TRK TKIs with strong potency against TRK resistance mutations are currently being explored in the clinic [35]. Among these, taletrectinib, while having mainly been evaluated as a ROS1 inhibitor in patients, has shown remarkable activity against TRK fusion-positive models [42]. However, in the case of the TRKA G667C xDFG mutant kinase, also this agent showed minimal efficacy, suggesting once again the potential superiority of zurletrectinib in this context. Importantly, proteolysis targeting chimeras (PROTACs) against TRK kinases have also begun testing. As these agents promote TRK degradation, they may potentially be equally active against both TRK WT and mutant kinases. However, limited data are currently available on the activity of these drugs in patients with TRK fusion-positive, TRK WT and mutant tumors. In addition, the larger size of PROTACs when compared to conventional TKIs often limits their brain penetration capacity [43], thus making them less suitable for targeting gliomas or brain metastasis.

In summary, zurletrectinib is a potent next-generation TRK inhibitor with stronger efficacy than other clinically developed next-generation agents against most TRK resistance mutations. It also shows increased brain penetration which results in better intracranial activity. While zurletrectinib’s activity and safety profile in patients are not yet fully determined, its preclinical features place it among the best-in-class next-generation TRK inhibitors for the treatment of *NTRK* fusion-positive, TRK mutated tumors.

### Supplementary information

Supplementary methods and figures

## Data Availability

Raw data are available upon request.
