# Supplementary material for: Zurletrectinib is a next-generation TRK inhibitor with strong intracranial activity against NTRK fusion-positive tumours with on-target resistance to first-generation agents
Source: Br J Cancer. 2024 Jun 20;131(3):601–10. doi: 10.1038/s41416-024-02760-1 (PMC11300601; doi:10.1038/s41416-024-02760-1)
Supplement: Supplementary file 1 — Supplementary methods and figures [file 41416_2024_2760_MOESM1_ESM.pdf]

Supplementary methods

Structural and interactional analysis between compounds and TRKA kinase
2D projections of the zurletrectinib-TRKA interactions were generated using LigPlot+[38]. Superimposition of zurletrectinib and repotrectinib in complex with TRKA wild type was generated using PyMol.

***In vitro* kinase assay, crystal violet, and western blot quantifications**
In vitro kinase assay quantification was performed using GraphPad Prism 9. Bar graphs displaying IC_{50}s ± STDEV of larotrectinib, selitrectinib, repotrectinib, and zurletrectinib against all recombinant kinases were generated using GraphPad Prism 9. Crystal violets were quantified by adding 400 uL of 33% acetic acid to each well. Plates were agitated on a rocking platform for 10 minutes. Once dissolved, samples were transferred in triplicates onto a 96 well plate. Absorbances were read at 590 nm and data was analyzed using GraphPad Prism 9. Optical densities (ODs) were plotted using GraphPad Prism 9 and results are presented as mean ± STDEV. Quantification of western blots were performed using ImageJ and bar graphs were generated using GraphPad Prism 9.

Drug screening on *Ntrk* fusion-positive mouse glioma cell lines
CellTiter-Glo Cell Viability Assays (Promega) and crystal violet clonogenic assays were performed on isogenic mouse glioma cell lines. For the CellTiter-Glo assays, three biological replicates were performed, with each condition being assayed in triplicate determinations. Cells were seeded in a 96-well plate in the afternoon at optimal density. The following morning, larotrectinib, selitrectinib, repotrectinib, zurletrectinib and cabozantinib (1:3 dilutions with a maximum concentration of 2.50 μM) were added. Plates were removed from the incubator 72 hours later and CellTiter-Glo reagent was added. Absorbance was read at 490 nm in accordance with Promega's protocol. Data is presented as a survival percentage on the y-axis (mean ± STDEV) normalized to the control DMSO-treated cells deemed 100% viable. Drug concentrations on the x-axis are represented as a base 10 logarithm (LOG). For the crystal violet assays, cells were seeded in a 24-well plate at optimal densities in the afternoon. The following morning, larotrectinib, selitrectinib, repotrectinib and zurletrectinib were added at concentrations ranging from 10 nM to 2,500 nM. Crystal violet plates were removed from the incubator 72 hours later, washed with PBS, fixed with 4% paraformaldehyde for 15 minutes, and stained with crystal violet for 10 minutes. Crystal violet was washed off and plates were left to dry prior to imaging.

Supplementary figures

Supplementary Figure 1

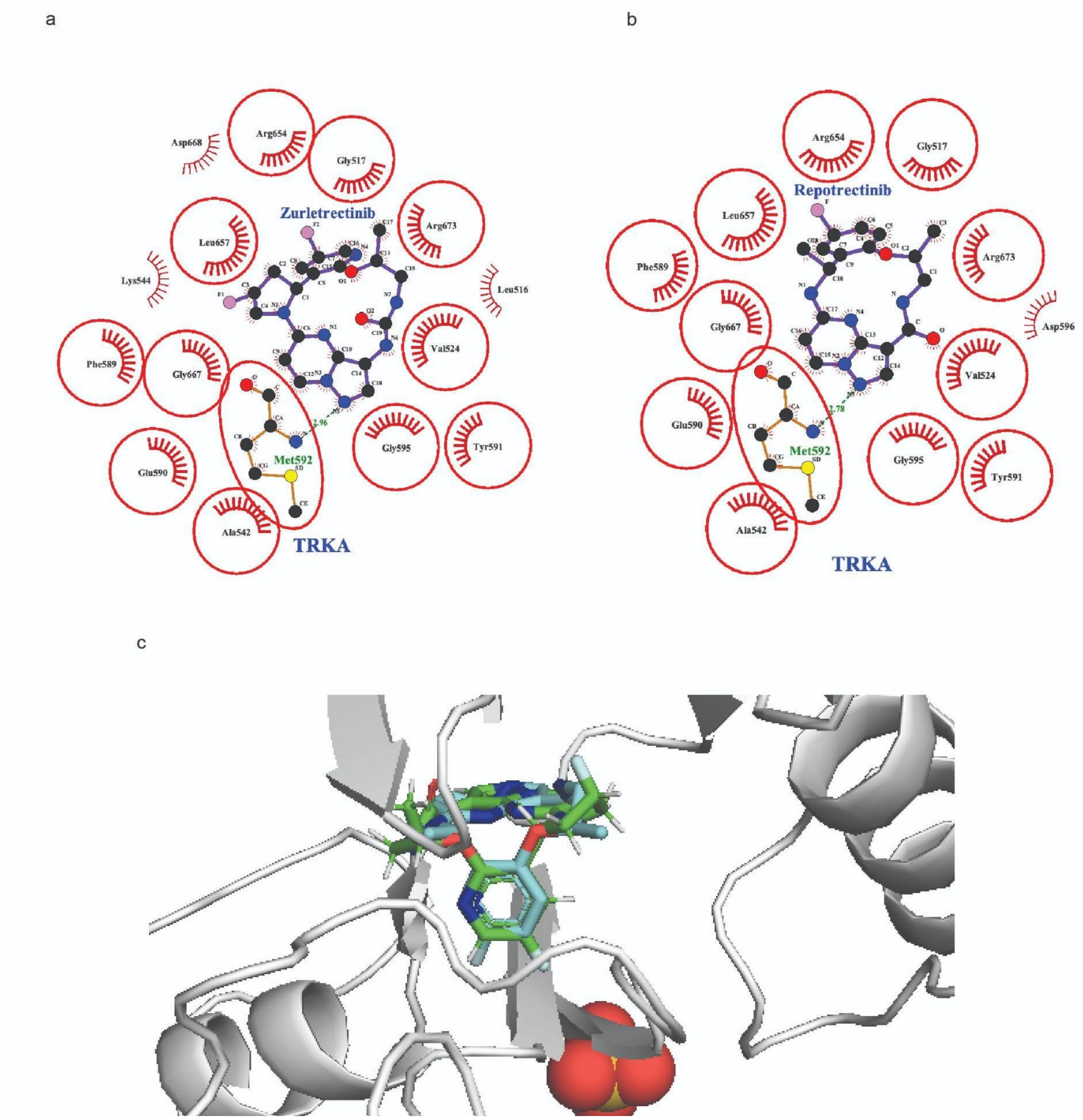

Supplementary Figure 1. **Interactions of zurletrectinib and repotrectinib with the TRKA kinase**

2D projections of the zurletrectinib-TRKA (**a**) and repotrectinib-TRKA (**b**) interactions are displayed. Shared interactions are circled in red. Red lines represent hydrophobic interaction while green lines hydrogen bonds. Graphs were generated using LigPlot+. (**c**) Superimposition of zurletrectinib (cyan) and repotrectinib (green) in complex with TRKA WT. The image was generated using PyMol.

Suppl Figure 2

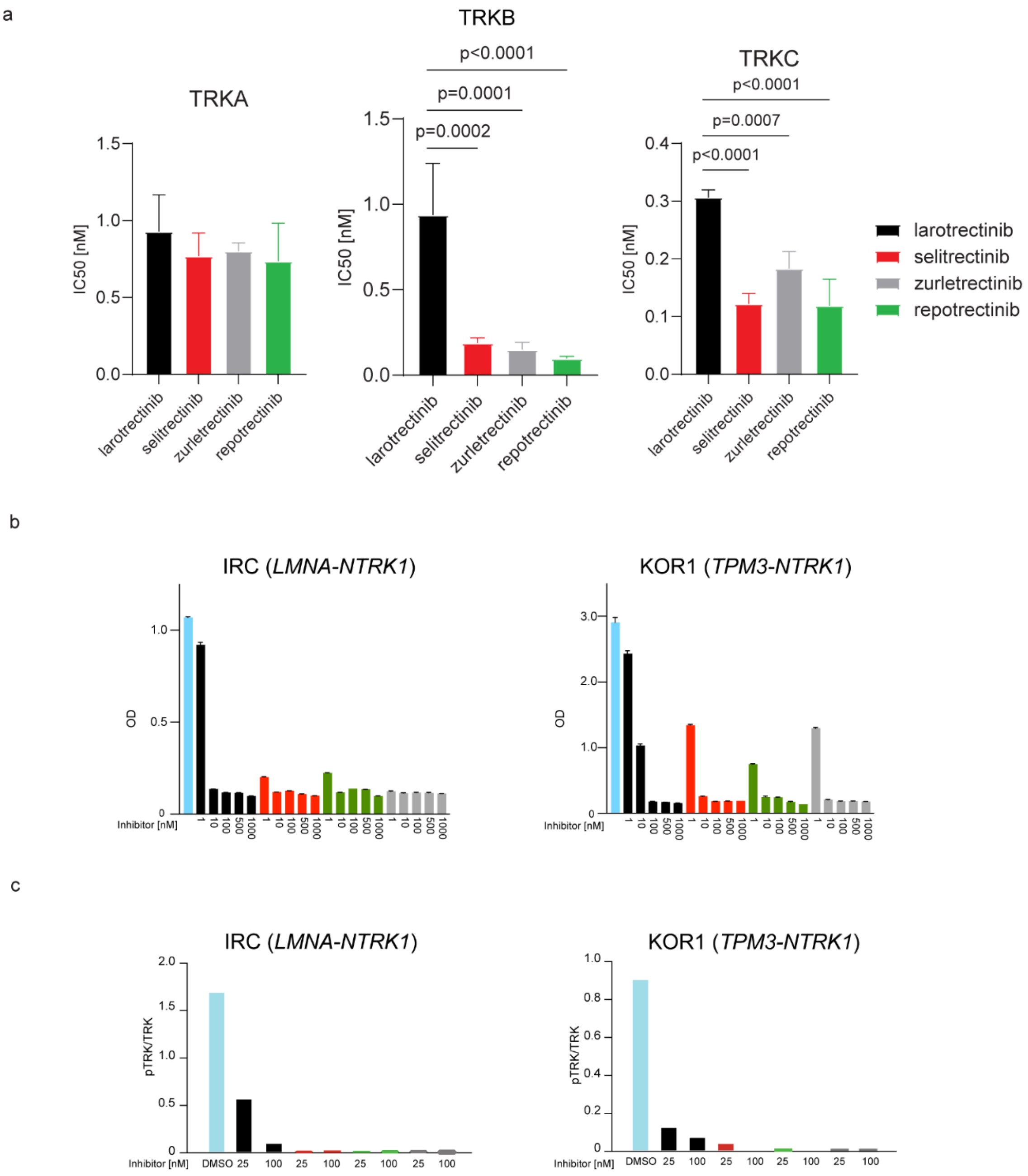

Supplementary Figure 2. **Quantification of assays presented in Figure 1**
(**a**) *In vitro* kinase assay quantification. Bar graphs displaying IC_{50}s of larotrectinib, selitrectinib, repotrectinib, and zurletrectinib against TRKA, TRKB, and TRKC recombinant kinases. (**b**) Quantification of clonogenic assays following acetic acid-based extraction of crystal violet. Each well was read in triplicate. Optical densities (ODs) were plotted using GraphPad Prism 9. Results are presented as mean ± STDEV. (**c**) Quantification of pTRK/TRK signals detected by western blot. Analyses were performed on a representative blot for each cell line using ImageJ and bar graphs were generated using GraphPad Prism 9.

Suppl Figure 3

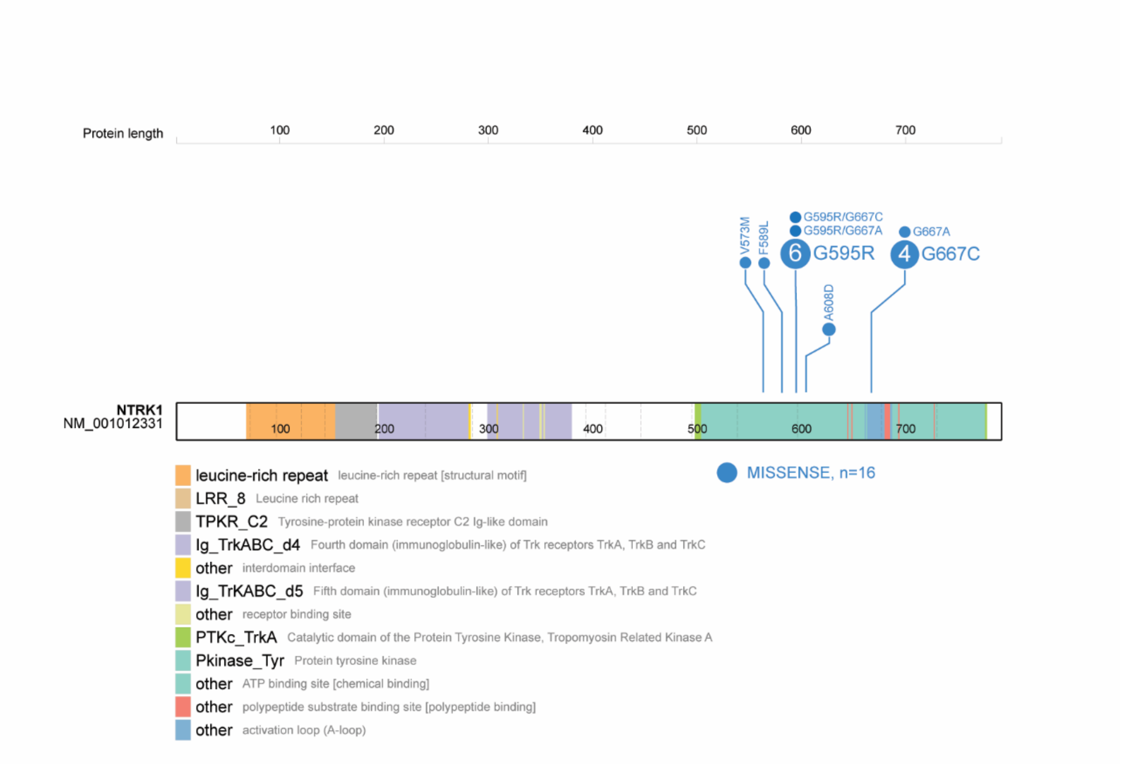

Supplementary Figure 3. TRK inhibitor resistance mutations in TRKA
Schematic depicting TRKA resistance mutations identified in patients progressing to 1st- and/or next-generation TRK inhibitors. The different domains of the TRKA kinase are indicated.

Suppl Figure 4

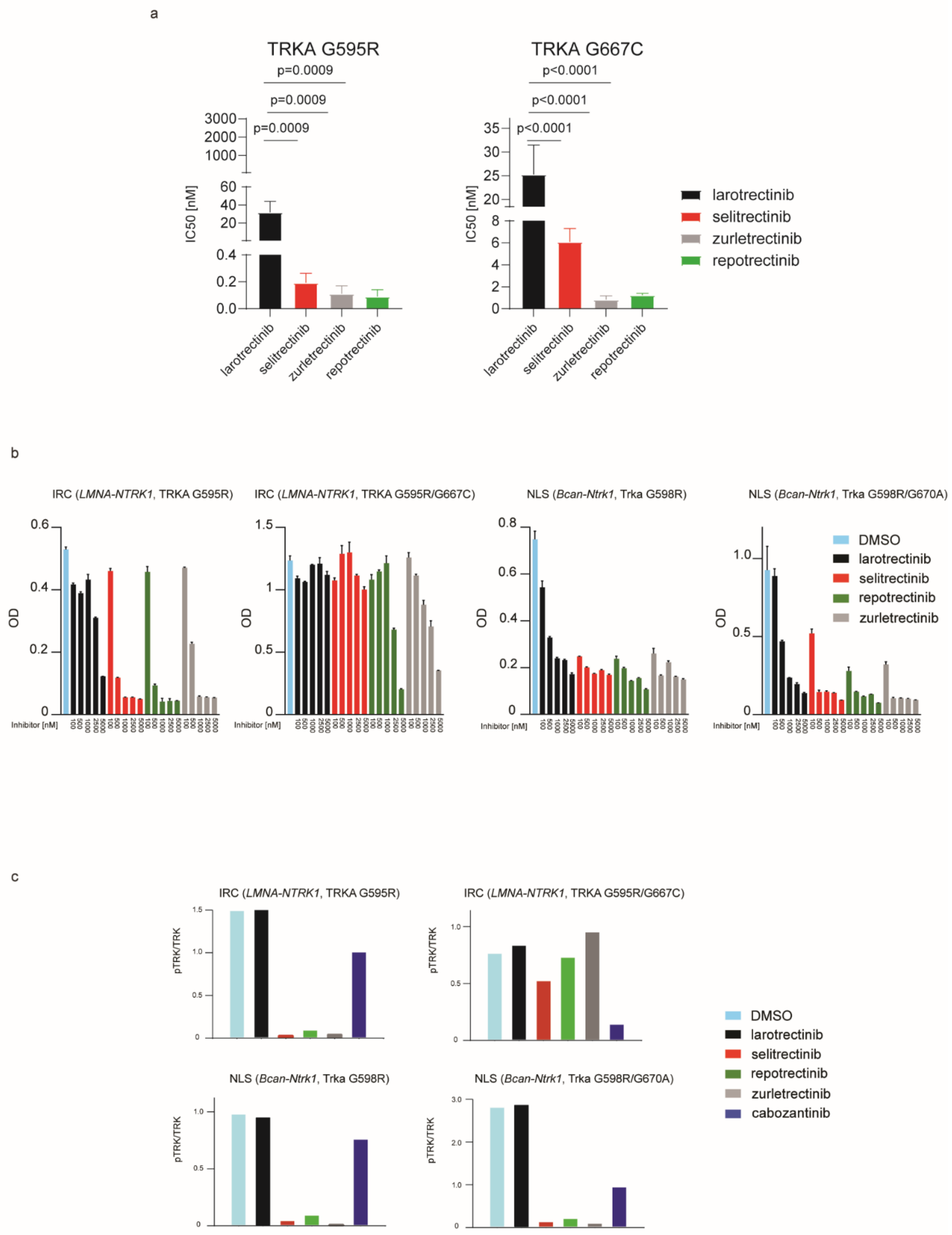

Supplementary Figure 4. Quantification of assays presented in Figure 2

(**a**) *In vitro* kinase assay quantification. Bar graphs displaying IC_{50}s of larotrectinib, selitrectinib, repotrectinib, and zurletrectinib against TRKA mutant recombinant kinases. (**b**) Quantification of clonogenic assays following acetic acid-based extraction of crystal violet. Each well was read in triplicate. Optical densities (ODs) were plotted using GraphPad Prism 9. Results are presented as mean ± STDEV. (**c**) Quantification of pTRK/TRK signals detected by western blot. Analyses were performed on a representative blot for each cell line using ImageJ and bar graphs were generated using GraphPad Prism 9.

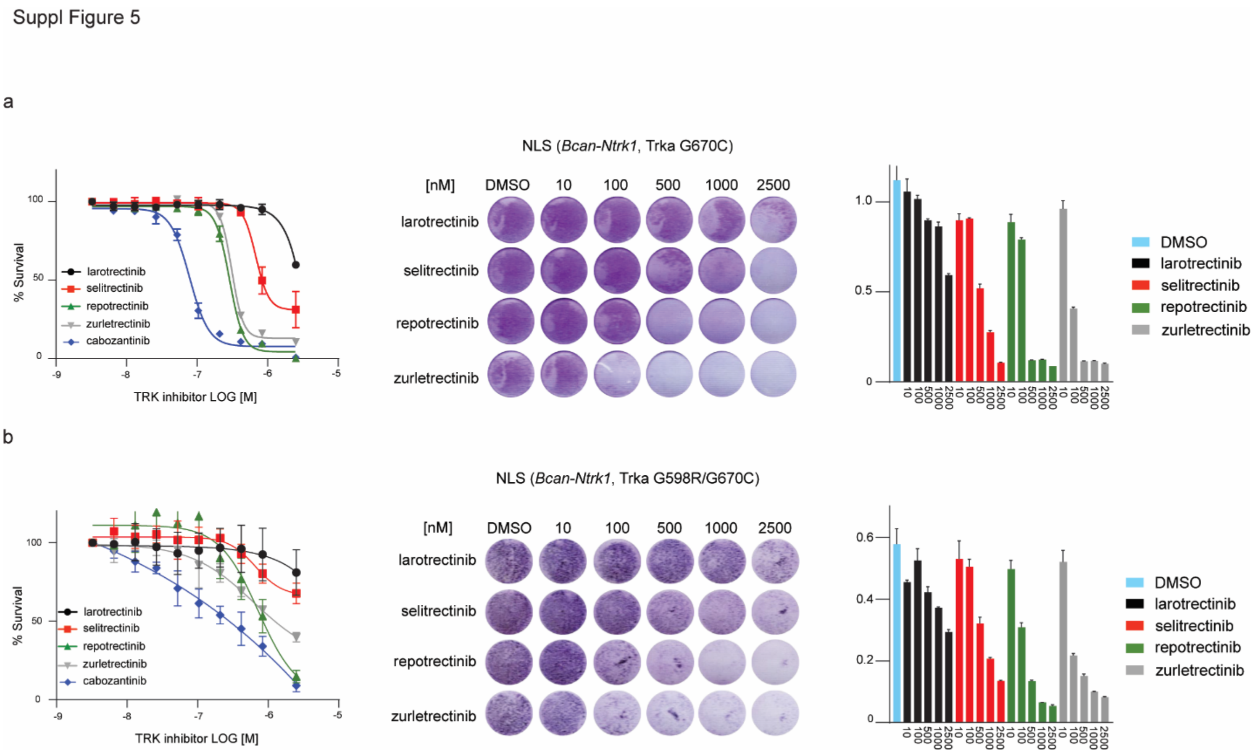

Supplementary Figure 5. Activity of zurletrectinib against Trka G670C and G598R/G670C mutant mouse *Bcan-Ntrk1* positive glioma cell lines

(**a**, **b**) Proliferation and clonogenic assays, and western blot analyses following treatment of *Ntrk* fusion-positive mouse glioma cell lines harboring the Trka G670C single (**a**) or the Trka G598R/G670C double (**b**) mutations with zurletrectinib. The activity of zurletrectinib was compared against the activity of other 1st- (larotrectinib) or next-generation (selitrectinib and repotrectinib) TRK inhibitors. Cabozantinib was added in the Cell-Titer Glo-based assays as control.

Suppl Figure 6

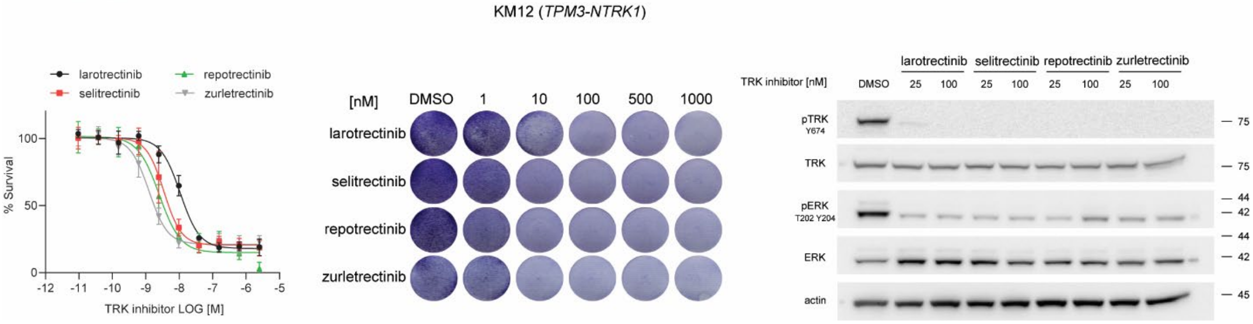

Supplementary Figure 6. *In vitro* activity of zurletrectinib against the *TPM3-NTRK1* CRC cell line KM12

Proliferation and clonogenic assays, and western blot analyses following treatment of the *NTRK* fusion-positive cancer cell line KM12 with zurletrectinib. The activity of zurletrectinib was compared against the activity of other 1st- (larotrectinib) or next-generation (selitrectinib and repotrectinib) TRK inhibitors.
